# Stem Cell Therapy for Spinal Cord Injury: A Review of Recent Clinical Trials

**DOI:** 10.7759/cureus.24575

**Published:** 2022-04-28

**Authors:** Emmanouil I Damianakis, Ioannis S Benetos, Dimitrios Stergios Evangelopoulos, Aikaterini Kotroni, John Vlamis, Spyridon G Pneumaticos

**Affiliations:** 1 Physical Medicine and Rehabilitation, KAT Hospital, Athens, GRC; 2 3rd Department of Orthopaedic Surgery, National and Kapodistrian University of Athens (NKUA) School of Medicine, KAT Hospital, Athens, GRC

**Keywords:** paraplegia, tetraplegia, functional recovery, clinical trial, stem cells, cell therapy, spinal cord injury

## Abstract

Spinal cord injury (SCI) remains an incurable, life-changing neurological condition, causing permanent loss of motor and sensory function in millions of people worldwide and affecting them in every aspect of their personal and social life. In the last two decades, after its success in various fields of medicine, stem cell therapy has been investigated in the research field as a potential treatment for SCI. This review focuses on the pathophysiology of SCI, the characteristics of the different stem cell therapies used for its treatment, and the results of these therapies in recently published clinical trials.

## Introduction and background

Every year, approximately 500,000 new incidents of spinal cord injury (SCI) occur around the world. In most cases, the cause is trauma due to automobile accidents, falls, gunshots, or medical/surgical complications [1]. Due to the nature of its causes, SCI is still a condition that affects mostly young people. However, as we live in a continuously aging society, rising numbers of new cases of SCI in the elderly population after low energy trauma are also seen [2]. The sequelae of pathophysiological events after the injury usually result in permanent neurological deficits, such as loss of motor and sensory function below the injury level and autonomic dysfunction. Furthermore, patients with SCI usually have to deal with socio-economic problems, especially if they live in countries where opportunities and social support for disabled people are poor.

Current clinical practice focuses on early surgical decompression and mechanical stabilization of the SCI site, followed by a pharmacological intervention such as methylprednisolone, nimodipine, naloxone, and others [3,4]. After the first critical phase, patients participate in rehabilitation programs to regain functionality and autonomy. Unfortunately, all these interventions have demonstrated poor outcomes regarding neuroprotection, neuroregeneration, and functional recovery. The reason behind this failure lies in the complexity of the pathophysiological mechanisms of SCI, which result in irreversible damage to the neuronal environment at the site of the injury.

In the last decades, stem cell therapy has emerged as a very promising solution in the field of SCI. After a plethora of encouraging treatment trials using different types of stem cells in animals of different species [5], clinical trials involving humans with SCI became a reality in the middle of the first decade of this century. This review addresses the pathophysiology of SCI, the possible mechanisms of action of different stem cells in spinal cord repair, and it presents the basic characteristics and results of the related clinical trials published in the PubMed online library in the last five years.

Pathophysiology of SCI

Primary and Secondary Injury

SCI pathophysiology can be divided into two phases. The first one, called primary injury, happens at the time that the mechanical force is posed on the spinal cord, causing compression, laceration, distraction, or shearing to the neuronal tissue, also damaging the blood vessels and the surrounding tissues. Studies have shown that primary injury almost never completely disrupts the anatomical continuity of the spinal cord [6].

Secondary injury can be further divided into four phases: immediate (0 - 2 hours after the injury), acute (early acute phase: 2 - 48 hours, subacute: 2 days - 2 weeks), intermediate (2 weeks - 6 months), and chronic phase (>6 months). The first detectable change is a generalized swelling of the spinal cord accompanied by hemorrhage in the central gray matter. The direct disruption of cell membranes and the ischemia resulting from vascular damage result in cell necrotic death, which leads to hemorrhage in the white matter [7,8] and produces cord ischemia that may extend to many spinal segments. In this phase, pro-inflammatory cytokines such as tumor necrosis factor-alpha (TNFα) and interleukin 1 beta (IL-1β) are upregulated and inflammation begins. Secondary processes such as ionic dysregulation, glutamate excitotoxicity, and immune neurotoxicity exacerbate axonal death. Radical-mediated lipid peroxidation contributes to axonal disruption. The disruption of the blood-spinal cord barrier induces neuroinflammation, which contributes further to neural cell death. In the subacute phase, astrocytes at the periphery of the lesion become hypertrophic, proliferate, and grow multiple large cytoplasmic processes. These cytoplasmic processes combine to form the gliotic scar. In the following intermediate phase, the gliotic scar matures. Finally, in the chronic phase of injury, Wallerian degeneration diminishes the injured axons and cystic cavitation is completed.

Prohibitors of Neuroregeneration

Glial scar: The formed glial scar has beneficial but also harmful effects [9]. On one hand, its components restore the blood-brain barrier by reducing cellular degeneration and inflammatory responses [10]. On the other hand, glial scar develops into a membrane that hinders axonal re-growth [11].

Microglia: This cell type is inactive in a healthy spinal environment [12]. In response to injury, the microglial cells become activated into phagocytes, exhibit chemotaxis and secrete cytokines, growth factors, chemokines, and neurotrophins. Their mission is to clear the cellular debris and toxic byproducts [13-16]. Neuronal death is caused by the microglial release of numerous pro-inflammatory cytokines, nitric oxide (NO), glutamate, and activation of reactive oxygen species (ROS) pathways from the injury site.

Myelin debris: During the demyelination process after the injury, high amounts of myelin debris accumulate on site. Some of the myelin proteins like myelin-associated glycoprotein (MAG), oligodendrocyte-myelin glycoprotein (OMgp), ephrin B3, semaphorin 4D (SEMA4D/CD100), while having a beneficial role in the initial development of the nervous system, in the event of injury, they exert an inhibitory effect on axonal regrowth [17-21].

Chondroitin sulfate proteoglycans (GSPGs): These proteoglycans have an active role in cell migration and axonal growth during the development of the CNS [22]. During SCI, these molecules are upregulated and accumulate on site. This contributes to the formation of the glial scar, which poses a mechanical barrier and creates a hostile environment for axonal growth.

## Review

Study design

**Figure 1:**
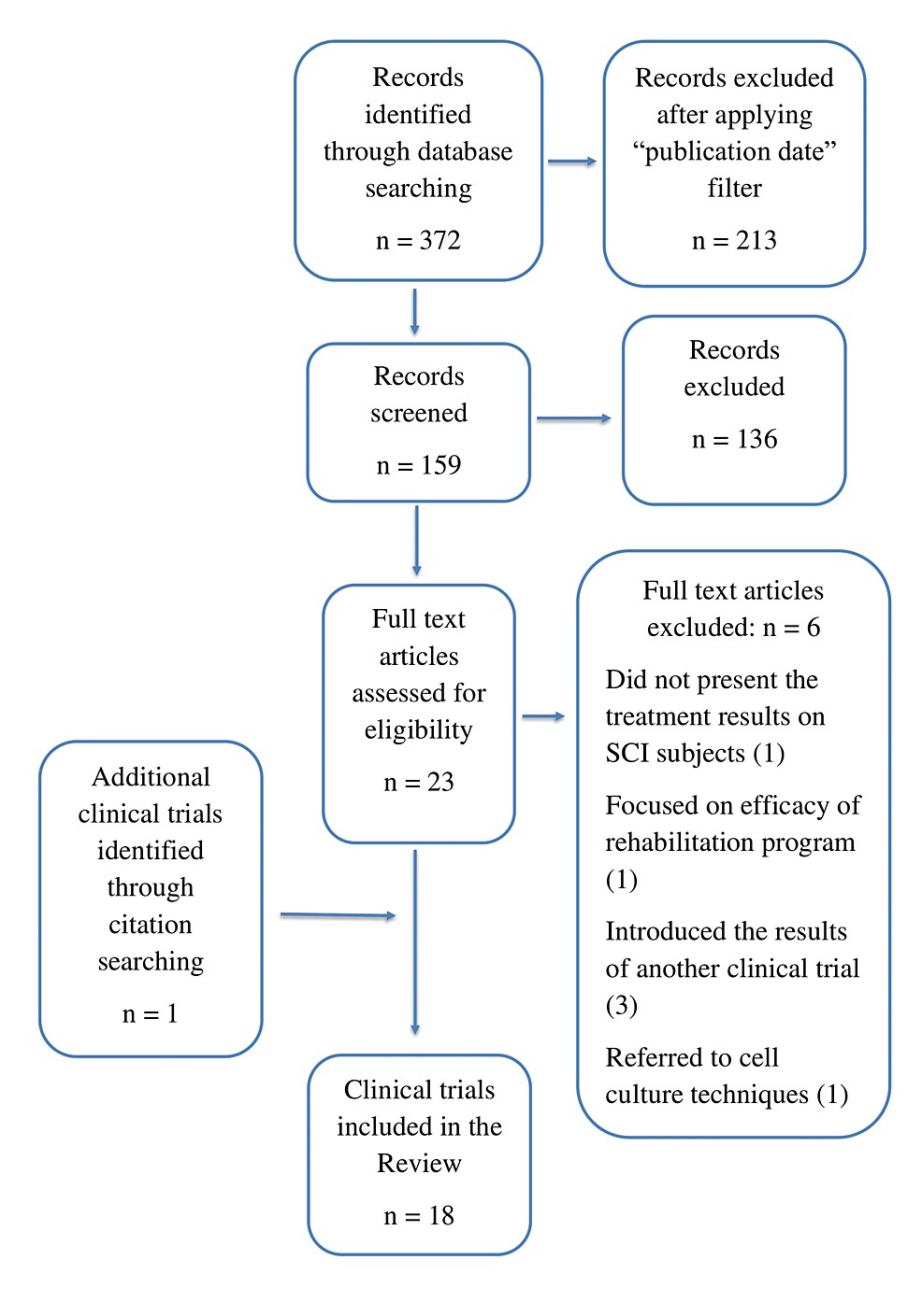
Selection process

The aim of this study was to review the published clinical trials using stem cells for the treatment of SCI from January 1, 2017, until March 31, 2022. For this purpose, a literature search was performed according to PRISMA guidelines, using the online PubMed database and the following keywords: spinal cord injury, cell therapy, stem cells, clinical trial. Inclusion criteria were: clinical trials using stem cells for SCI patients, focusing on the efficacy of treatment. An initial search resulted in 372 articles. After applying the "publication date" filter, 213 articles were excluded. After checking titles and abstracts, 136 more articles were rejected for not meeting inclusion criteria. A total of 23 articles were assessed for eligibility and six of them were excluded for the following reasons: one article did not present the treatment results of SCI subjects, one article focused on the efficacy of the rehabilitation program, three articles introduced the results of another clinical trial, and one article referred to cell culture techniques. An additional citation search returned one clinical trial that met the inclusion criteria, resulting in a total of 18 clinical trials included in the present review. The selection process is shown in Figure 1.

Overview of clinical trials and stem cells used

Table 1 summarizes reviewed clinical trials by cell type and year of publication.

**Table 1:** Summary of clinical trials ADL: activities of daily living, AIS: the American Spinal Injury Association Impairment Scale, BMSC: bone marrow–derived mesenchymal stromal cell, EMG: electromyography, FIM: functional independence measure, GRASSP: graded redefined assessment of strength, sensation and prehension, HUCBC: human umbilical cord blood cell, huCNS-SC: human central nervous system stem cell, IANR-SCIRFS: International Association of Neural Restoration Spinal Cord Injury Functional Rating Scale, ISCSCI-92: International Standards for Neurological and Functional Classification of Spinal Cord Injury, MEP: motor evoked potential, MRC: Medical Research Council's scale, NCS: Nerve Conduction Studies, NSC: neural stem cell, OEC: olfactory enseathing cell, QoL: quality of life, SC: Schwann cell, SEP: somatosensory evoked potential, SCIM: Spinal Cord Independence Measure, UCMSC: umbilical cord–derived mesenchymal stromal cell, WJ: Wharton jelly

Author	Phase of clinical trial	Cell origin	Cell type	Dose	Administration route	Number of patients	AIS classification or level of injury	Time from Injury (acute/subacute/chronic phase)	Follow up	Main outcome (functional improvement/adverse effects)
Honmou et al. [23] 2021	Phase II	Autologous	BMSC (auto-serum expanded)	84 − 150 × 10^6^	Intravenous	13	AIS A: 6, B: 2, C: 5	Subacute	6 months	AIS A→B (3/6 patients), A→C (2/6), B→C (1/2), B→D (1/2), C→D (5/5). Functional improvements according to ISCSCI-92 and SCIM-III scales.
Phedy et al. [24] 2019	Case report	Autologous	BMSC	10 − 17 × 10^6^ (× 7 times)	Intrathecal x1 Intravenous x6	1	AIS A T12	Chronic	5 years	AIS A→C. Increase in AIS score: 10→30. Increase in MRC score for L1 and L2 innervated muscles: 0/5→3/5.
Vaquero et al. [25] 2018	Phase II	Autologous	BMSC	100 × 10^6^ × 3 doses	Intrathecal	11	A: 3, B: 4, C: 3, D: 1	Chronic	10 months	AIS improvement in 27% of patients.
Vaquero et al. [26] 2018	Phase II	Autologous	BMSC	100 × 10^6^	Intramedullary	6	A: 3, B: 2, D: 1	Chronic	6 months	AIS improvement in 2 patients. Bowel and bladder function improved. 4 patients improved in NCS findings.
Guadala-jara et al. [27] 2018	Case report	Autologous	BMSC	300 × 10^6^ × 3 doses (1/month)	Intrathecal	1	AIS A T12	Chronic	6 months	Improvement in functionality and especially in Krogh's Neurogenic Bowel Dysfunction scale.
Vaquero et al. [28] 2017	Phase II	Autologous	BMSC	30 × 10^6^ × 4 doses	Intrathecal	10	Cervical, Thoracic, Lumbar	Chronic	12 months	AIS Improvement. Motor and sensory scores improved.
Deng et al. [29] 2020	Phase I	Allogeneic	UCMSC+ Scaffold	40 × 10^6^ (Collagen scaffold)	Intramedullary	20	AIS A Cervical	Acute	12 months	AIS A→B (9 patients), AIS A→C (2patients). Improvement in ADL scores. Improvement in bowel and bladder function.
Yang et al. [30] 2020	Phase I/II	Allogeneic	UCMSC	1 × 10^6^/kg (× 4 times)	Intrathecal	41	C1-C7: 24, T1-T9: 7, T10-L1: 10	Subacute or Chronic	12 months	Statistical increase in AIS and IANR-SCIRFS scores. Decrease in muscle spasticity. No serious adverse effects.
Xiao et al. [31] 2018	Phase I	Allogeneic	UCMSC+ Scaffold	40 × 10^6^	Intramedullary	2	AIS A C4, T11	Acute	12 months	AIS A→C in both patients.
Albu et al. [32] 2020	Phase I/IIa	Allogeneic	WJ-MSC	10 × 10^6^	Intrathecal	10	T3-T11	Chronic	6 months	Significant improvement in pinprick sensation in compared with placebo group. No changes in motor function, independence, QoL, SEPs, MEPs, spasticity or bowel function.
Curt et al. [33] 2020	Phase I/IIa	Allogeneic (Stemcells Inc.)	huCNS-SC®	20 × 10^6^	Intramedullary	12	A: 7, B: 5 (T2-T11)	Subacute or Chronic	6 years	Sensory improvements in 5 out of 12 patients. No motor improvements were observed.
Levi et al. [34] 2019	Phase I/II	Allogeneic (Stemcells Inc.)	huCNS-SC®	15 + 30 + 40 × 10^6^(Coh.I) 40 × 10^6^ (Coh.II)	Intramedullary	17 Cohort I:6, Cohort II:11 6/11 monitored	AIS A, B Cervical	Subacute or Chronic	12 months	Trial terminated prematurely by sponsor. Improvement in UEMS score. No adverse effects.
Levi et al. [35] 2018	Phase I/II	Allogeneic (Stemcells Inc.)	huCNS-SC®	15 − 40 × 10^6^	Intramedullary	29	Cervical: 17 (Cohort I: 6, Cohort II: 11) Thoracic: 12	Subacute	Up to 56 months	Improvement in AIS motor scores. 15 serious adverse effects in cervical group and 4 in thoracic.
Curtis et al. [36] 2018	Phase I	Allogeneic (Seneca Biopharma)	NSI-566®	6 injections (Mean number)	Intramedullary	4	AIS A Thoracic	Chronic	60 months	Improved AIS scores, neurological levels and EMG findings. No improvement in QoL. No serious adverse effects.
Zamani et al. [37] 2022	Phase I	Autologous	OEC+ BMSC	15 × 10^6^, OEC/BMSC = 1/1	Intrathecal	3	AIS A	Chronic	24 months	AIS A→B: 1 and 6 points improvement in SCIM. Mild adverse effects.
Gant et al. [38] 2021	Phase I	Autologous (sural nerve)	SC	50 − 200 × 10^6^ (Depended on patient’s cyst volume)	Intramedullary	8	Cervical: 4 Thoracic: 4	Chronic	5 years	The neurological level improved by 1 level in 1 patient. Improvement in Sensory score in all patients with thoracic and in 2 patients with cervical lesion.
Anderson et al. [39] 2017	Phase I	Autologous (sural nerve)	SC	5, 10 or 15 × 10^6^	Intramedullary	6	Thoracic	Subacute	12 months	AIS A→B:1. Improvement in FIM and SCIM III scores. No adverse effects.
Smirnov et al. [40] 2022	Phase I/IIa	Allogeneic	HUCBC	14.8 × 10^6^/kg (Total cell number for 4 infusions)	Intravenous	10	A: 6, B: 4	Acute (within 3 days of Injury)	12 months	No serious adverse effects related to therapy. AIS A→C: 3, AIS B→D: 2, AIS B→E: 2, AIS A→D: 1

Mesenchymal stem cells

Mesenchymal cells are multipotent progenitor cells that can be easily isolated from various tissues such as bone marrow, adipose tissue, umbilical cord, and amniotic fluid. Their use does not raise ethical concerns. Furthermore, their profile is of low tumorigenicity and immunoreactivity [41,42]. They are believed to act at the site of the lesion through the secretion of multiple factors, such as growth and adhesion factors, anti-inflammatory factors, and cytokines. Thus, their effects are considered anti-apoptotic, neurotrophic, neuroprotective, and immunomodulatory [22,43].

Bone Marrow-derived Mesenchymal Stem Cells

Bone marrow-derived mesenchymal stem cells (BMSCs) are harvested from the bone marrow, they are multipotent and can be differentiated into various cell types, including osteoblasts, fibroblasts, chondroblasts, chondrocytes, adipocytes, neurons, and glial cells. Their beneficial attributes seem to emerge through their immunosuppressive and neuroprotective roles and are due to the numerous growth factors released at the site of lesions, such as NGF (nerve growth factor), VEGF (vascular endothelial growth factor), GDNF (glial-derived neurotrophic factor), BDNF (brain-derived neurotrophic factor), NT-3 (neurotrophin-3), FGF (fibroblast growth factor), and EGF (epidermal growth factor) [44].

Six clinical trials using BMSCs were identified in the literature search. Four of them are in phase II and two of them have been published as case studies. A total of 42 patients received this type of stem cell therapy. In four studies, the administration was intrathecal, in one study it was intravenous, and in another one, the cells were delivered with a combination of intravenous and intrathecal methods. Out of the 42 patients, 13 were in the subacute stage of the injury at the time of the transplantation and the rest were in the chronic stage.

Honmou et al. [23] in 2021 treated 13 patients in the subacute phase of SCI with autologous BMSCs. Six of them were classified as AIS A injuries, two as AIS B and five as AIS C. The cells were expanded in autologous serum, and the dose was between 84 and 150 million cells. There was one intravenous delivery of cells, and the patients were followed up for six months. The results were promising, showing an improvement in AIS classification in seven patients as well as in functional activities according to the ISCSCI-92 and SCIM III scales.

In a case report published by Phedy et al. [24] in 2019, one patient with T12 level injury was administered one intrathecal dose and six intravenous doses of BMSCs. Each dose contained 10-17 million cells, and the duration of the follow-up was five years. At the end of the follow-up, there was an improvement in the patient’s AIS score, as well as in the MRC score for L1 and L2 innervated muscles bilaterally.

Vaquero et al. [25] published the results of a phase II clinical trial in 2018. A total of 11 patients in the chronic phase of SCI were included in the trial. Each of them received three doses of intrathecal administration of 100 million BMSCs and was followed for 10 months. The final results showed an improvement in AIS classification in 27% of the patients.

In another published phase II clinical trial, Vaquero et al. [26] administered 100 million BMSCs in a single dose to six chronic SCI patients. Three of them were classified as AIS A, two as AIS B, and one as AIS D. After six months, bowel and bladder management improved in some patients. Four out of six patients had better NCS measurements, while two of them improved in AIS classification.

A case report by Guadalajara et al. [27] in 2018 introduced a patient with chronic AIS-A T12 level injury. The patient received autologous BMSC in three doses (one every month). Each dose consisted of 300 million BMSCs. After six months, significant improvement in the patient’s functionality was observed, especially on Krogh's Neurogenic Bowel Dysfunction scale.

In 2017, Vaquero et al. [28] published the results of a phase II clinical trial that included transplantation of autologous BMSCs in 10 patients. The patients had chronic injuries to the cervical, thoracic, or lumbar spinal cord. They were administered four doses of 30 million BMSCs and were monitored for 12 months. The results were encouraging, as motor and sensory scores improved.

Umbilical Cord Mesenchymal Stem Cells

Umbilical cord mesenchymal stem cells (UCMSCs) are harvested from the human umbilical cord. They can be easily expanded in vitro and have low immunoreactive properties. They produce numerous factors, such as cytokines, growth factors, interleukins, BDNF, bFGF (basic Fibroblast Growth Factor), and neutrophil activators. It is considered that these cells exhibit anti-inflammatory, anti-apoptotic, neurotrophic, and proangiogenic effects when transplanted [44].

In this review, three clinical trials using UCMSCs were identified. In two of them, UCMSCs were combined with scaffolds. Scaffolds are highly porous biomaterials prepared for biocompatibility that act as templates for tissue regeneration and potentially guide the growth of new tissue. In these clinical trials, 63 patients participated during the acute, subacute, or chronic stages of SCI.

Deng et al. [29] transplanted intramedullary 40 million cells combined with collagen scaffold in 20 patients with cervical injury (AIS A). The scaffold was prepared from bovine aponeurosis and was seeded with 40 million UCMSCs. The patients received the transplants and were followed for 12 months. A control group received conventional treatment (infection prevention and supportive treatment). After 12 months, nine of the transplanted patients converted from AIS A to B and two from AIS A to C. Improvement in ADL scores and bowel/bladder management were also observed, compared to the control group.

In 2020, Yang et al. [30] published the results of the a phase I/II clinical trial. A total of 41 patients with subacute or chronic injuries were included. They received four intrathecal injections of one million UCMSCs per injection and were monitored for 12 months. No adverse effects were recorded. The results showed an increase in AIS and IANR-SCIRFS scores and a decrease in muscle spasticity.

Xiao et al. [31] in 2018 transplanted intramedullary 40 million UCMSCs combined with a NeuroRegen collagen scaffold in two acute SCI patients (AIS A). The scaffold was prepared from bovine aponeurosis. The patients were followed for six months. At the end of the follow-up, both patients had converted from AIS A to C and no adverse effects were recorded.

Umbilical Cord Wharton-Jelly Mesenchymal Stem Cells

Wharton jelly is a gelatinous substance within the umbilical cord that insulates the blood vessels. Wharton-Jelly mesenchymal stem cells (WJ-MSCs) can be extracted and cultured. They produce a variety of neurotrophic factors and can promote neurogenesis and angiogenesis due to the release of significant levels of NGF, bFGF and GDNF. It has been shown in animals that repeated infusion of WJ-MSCs can decrease glial scar and it can cause regeneration of the spinal cord.

Albu et al. [32] used WJ-MSCs to treat 10 patients with chronic SCI (T3-T11) in a phase I/II, RCT, placebo controlled clinical trial. The patients received the cells via intrathecal delivery and were followed for six months. In the end of the follow up, significant improvement in pinprick sensation was noted compared to the placebo group, while no change in motor function, independence measurements, QoL, SEPs, MEPs, spasticity or bowel function was observed.

Neural stem cells

Neural stem cells (NSCs) have the potency to differentiate into glia, neurons, and astrocytes. They have been located in the dentate gyrus of the hippocampus, the lateral brain ventricle, and the spinal cord central canal [45]. Studies have shown that NSCs secrete growth factors that help damaged cells survive [46]. They enhance oligodendrocyte differentiation, thus providing a supportive role in remyelination [47]. They also enhance neural differentiation, replace necrotic neurons, make synapses in the gray matter, and restore functionality [48]. Finally, they act as a barrier, protecting against the enlargement of the initial lesion area [49].

Human Central Nervous System Stem Cells and NSI-566®

HuCNS-SCs® is a fetal brain-derived human central nervous system stem cell progenitor population. They are purified by surface marker expression, expanded, and then banked. HuCNS-SCs® have shown neuroprotection abilities, and they have the advantage of being non-tumorigenic compared to embryonic stem cells. Several clinical trials using HuCNS-SCs for Pelizaeus-Merzbacher disease, age-related macular degeneration (AMD), and neuronal ceroid lipofuscinosis have been completed without safety issues and with a good survival rate of transplanted cells [50,51].

NSI-566® is a human spinal cord-derived neural stem cell line that originates from the spinal cord of an eight-week-old aborted fetus. They have been used in clinical trials for the treatment of ALS. When injected into the spinal cord, they differentiate into neurons, support the impaired ALS motor neurons, and form connections. Their beneficial role lies in the secretion of multiple neurotrophic factors.

Since the beginning of 2017, four clinical trials using neural progenitor cells derived from human fetal CNS have been identified. Three of the patients were administered HuCNS-SC® and one clinical trial used NSI-566®. A total of 62 patients received the cells intermedullary.

Curt et al. [33] in 2020 used HuCNS-SC® to treat 12 patients with subacute or chronic thoracic injuries in a phase I/IIa clinical trial. All patients received a single dose of 20 million cells intermedullary and were monitored for six years. The final results showed only sensory improvement in some patients (5/12).

In 2019, Levi et al. [34] published the preliminary results of a phase I/II clinical trial that was prematurely terminated by the sponsor. The original trial was designed to enroll a total of 52 patients in cohorts I, II, and III. The trial was terminated during the second cohort. In cohort I, six patients received 15, 30, or 40 million cells, with two participants in each dose assignment. All of them were followed for 12 months and were evaluated with Upper Extremity Motor Score (UEMS), GRASSP, and MRI. In cohort II, out of 25 participants, 13 were randomized to treatment and 12 to the control group. For the treatment group, only 11/13 received the transplantation (40 million cells) prior to trial termination. There is sufficient data from the follow-up of 10/25 patients. The analysis showed a trend toward UEMS and GRASSP motor gains, but at a lower magnitude than that set by the sponsor. This resulted in early study termination.

In another clinical trial (phase I/II), Levi et al. [35] treated 29 patients with subacute cervical or thoracic spinal cord injuries with intermedullary administration of neural progenitor cells. In phase I/II open-label, 12 subjects with a T2-T11 received a single dose of 20 million HuCNS-SCs®. The patients were followed for up to 56 months. A total of 31 patients were enrolled in the cervical study that included cohorts I and II (with controls), and two patients were nonrandomized due to early study termination. The cervical injury group was followed for 12 months. Cervical cohort I included two subjects for each of three escalation dosages, for a total of six subjects. Group Ia received four injections in two lesion sites (15 million cells in total), group Ib received six injections (30 million cells), and group Ic, eight injections (40 million cells). Each group consisted of two patients. Cohort II included 11 patients that received a single 40 million cell dose. The results showed improvement in AIS motor scores. There were four serious adverse events in four participants with thoracic SCI (cerebrospinal fluid leakage, constipation, and urinary tract infection) and 15 serious adverse events in nine participants with cervical SCI (cerebrospinal fluid leakage, constipation, autonomic dysreflexia, postprocedural sepsis, posterior reversible encephalopathy syndrome, constipation, seizure, urinary tract infection, wound hematoma, and aphasia), although no safety concerns were considered related to the cells or the manual intramedullary injection.

Curtis et al. [36] in 2018 published the results of a phase I clinical trial of four patients with chronic thoracic AIS A SCI who received a mean number of six intermedullary injections of NSI-566®. The patients were monitored for 60 months and the results were promising, showing an improvement in neurological levels (T8→T10 and T5→T7) and AIS motor and sensory scores in two patients, improved EMG findings in three of them, but no significant differences in QoL. Magnetic resonance imaging (MRI) and diffusor tensor imaging (DTI) showed no visible morphologic changes and no adverse effects were noted.

Olfactory enseathing cells

Olfactory enseathing cells (OECs) are specialized glial cells that are found in the olfactory epithelium in the nose and in the brain olfactory bulb. They can be harvested relatively easily by a minimally invasive endoscopic surgery. They secrete growth factors and can remyelinate large axons. They can also play a significant role by inducing signals necessary for cell guidance in order to achieve regeneration. Finally, they seem to regulate astrocyte activity [52].

In a phase I clinical trial published in 2022, Zamani et al. [37] used a combination of autologous OECs and BMSCs intrathecally (15 million cells, 1:1 ratio) to treat three patients with chronic AIS A SCI. The patients followed a rehabilitation program before the transplantation and until dismissal, and they were monitored for 24 months. MRI, SCIM III, and the AIS impairment scale were used to assess the outcome. In one patient, AIS classification was improved (AIS A→B) and six points of elevation were noted in SCIM III, regarding self-care and bed mobility. The other two patients showed no change in AIS and SCIM III scores. Two patients reported urination and defecation sensations but no bladder or bowel sphincter control. No serious adverse effects were reported, but two of the patients complained of neuropathic pain and one reported increased spasticity. All adverse effects were alleviated with the proper treatment without problems.

Schwann cells

Schwann cells (SCs) can be easily harvested from peripheral nerves, such as the sural nerve, and expanded in vitro. They secrete a number of growth factors and they can guide the regenerating axons [52]. It has also been demonstrated in various studies that they can support remyelination of the existing spared remyelinated axons [6].

Gant et al. [38] published a phase I clinical trial in 2021 where they used injections of SCs to treat eight patients with chronic AIS A injury (four cervical and four thoracic SCI). A sural nerve harvest was performed on every patient, and the cells were purified, preserved, and expanded for transplantation. The volume of the intramedullary cavity was calculated in each of the subjects based on preoperative screening, and they were filled with SCs. All patients followed pre and post-transplant rehabilitation programs. In the five-year follow-up, no serious adverse effects were related to the transplantation. Mild adverse effects concerning transplantation were headaches, nausea, and hypoesthesia. Improvements in sensory scores were noted in all patients with thoracic SCI and two patients from the cervical cohort. One patient transcended from AIS B to AIS C. Observed changes also included the emergence of motor evoked potentials (MEPs) and subclinical EMG activation in the legs and intercostals below the pre-treatment neurological level. In addition, changes indicating recovery of sympathetic activity were documented.

In 2017, a phase I trial was published by Anderson et al. [39]. In this trial, SCs were harvested from the sural nerve of six patients with subacute thoracic injury. The cells were transplanted with an injection into the epicenter of the lesion. Two of the participants received 5 million cells, two received 10 million cells, and the last two received 15 million cells. All patients were monitored for one year. No adverse effects were documented related to cell therapy. One patient converted from AIS A to B, gained nine sensory points, and showed detectable MEPs in the legs. Three subjects had voluntary EMG activation in their legs. All patients improved their FIM and SCIM III scores.

Human umbilical cord blood cells

The use of human umbilical cord blood cells (HUCBCs) is advantageous because of its ease of access and its non-invasive collection procedures. Human umbilical blood contains a large source of hematopoietic stem cells (HSCs) that are able to generate erythroid and myeloid progenitor colonies. Specifically, the proliferation capacity of human umbilical cord blood mononuclear progenitor cells (HUCBC) is much higher than that of similar cells in the bone marrow. They exhibit immunologic tolerance, are less reactive to human leukocyte antigen mismatch, and can therefore be used in allogeneic transplants with ease [53]. At the lesion site, HUCBCs seem to have a multifactorial contribution. They can differentiate into neural lineages, exhibit anti-apoptotic effects, induce angiogenesis and axonal remyelination, and express multiple growth factors [54].

In 2022, Smirnov et al. [40] published the results of a phase I/IIa clinical trial using HUCBCs via intravenous injections to treat 10 patients with acute SCI (AIS A or B). Four injections were administered to each patient within three days post-injury. The participants were followed for 12 months. No adverse effects related to cell therapy were reported. Five patients improved their AIS level by two points (A→C, B→D) and one patient by three points (B→E).

Discussion

Stem cell therapy for SCI is still in its infancy. Every clinical trial offers great knowledge in the field of research. However, standardization between studies still seems to be missing. Besides the stem cell type used, there is great heterogeneity regarding the preferred administration route, number of cells per dose, number of doses, and timing of the transplantation. This way, it is difficult for various trial results to be compared. Furthermore, SCI patients are an extremely variable population because of the heterogeneity of the injury. Their functional recovery can vary, especially when patients are still in the acute phase of the injury. Recruiting patients in the acute phase can make interpretation of functional outcomes extremely difficult, especially for stem cell trials that do not include appropriate control groups. Finally, there is still no consensus regarding the optimal follow-up duration that will ensure long-term adverse effects exposure. After all, tumorigenicity is a known and extremely serious adverse effect of some types of stem cells, and ectopic growth may take years to manifest after transplantation.

## Conclusions

Stem cell-based therapies offer an intriguing therapeutic potential for SCI. Combinational approaches with the use of biomaterials may also play an important role in improving SCI therapies. A deeper understanding of the pathophysiological mechanisms of SCI appears to be essential for the development of even more efficacious treatments than the ones already being investigated. Several concerns also need to be addressed, like ethical issues, tumorigenicity, immunogenicity, and immunotoxicity of various stem cells. Finally, enhanced funding seems to be necessary for the delivery of even more powerful and well-designed phase I and II clinical trials that will lead the way for the first phase III studies in the next few years.
